# Contribution of the KSHV and EBV lytic cycles to tumourigenesis

**DOI:** 10.1016/j.coviro.2018.08.014

**Published:** 2018-10

**Authors:** Oliver Manners, James C Murphy, Alex Coleman, David J Hughes, Adrian Whitehouse

**Affiliations:** 1 School of Molecular and Cellular Biology, University of Leeds, Leeds, LS2 9JT, United Kingdom; 2 Astbury Centre for Structural Molecular Biology, Faculty of Biological Sciences, University of Leeds, Leeds, LS2 9JT, United Kingdom; 3 School of Biology, Biomolecular Sciences Building, University of St Andrews, Fife, KY16 9AJ, United Kingdom; 4 Department of Biochemistry & Microbiology, Rhodes University, Grahamstown, 6140, South Africa

## Abstract

Kaposi’s Sarcoma-associated herpesvirus (KSHV) and Epstein Barr virus (EBV) are the causative agents of several malignancies. Like all herpesviruses, KSHV and EBV undergo distinct latent and lytic replication programmes. The transition between these states allows the establishment of a lifelong persistent infection, dissemination to sites of disease and the spread to new hosts. Latency-associated viral proteins have been well characterised in transformation and tumourigenesis pathways; however, a number of studies have shown that abrogation of KSHV and EBV lytic gene expression impairs the oncogenesis of several cancers. Furthermore, several lytically expressed proteins have been functionally tethered to the angioproliferative and anti-apoptotic phenotypes of virus-infected cells. As a result, the investigation and therapeutic targeting of KSHV and EBV lytic cycles may be essential for the treatment of their associated malignancies.

**Current Opinion in Virology** 2018, **32**:60–70

This review comes from a themed issue on **Viruses and cancer**

Edited by **Cary Moody** and **Erle Robertson**

For a complete overview see the Issue and the Editorial

Available online 28th September 2018

**https://doi.org/10.1016/j.coviro.2018.08.014**

1879-6257/© 2018 The Authors. Published by Elsevier B.V. This is an open access article under the CC BY license (http://creativecommons.org/licenses/by/4.0/).

## Introduction

Kaposi’s sarcoma associated herpesvirus (KSHV) and Epstein Barr virus (EBV) are double stranded gammaherpesviruses which contribute to the oncogenesis of several human tumours. KSHV is the etiological agent of the endothelial cell tumour Kaposi’s Sarcoma, in addition to two lymphoproliferative disorders; primary effusion lymphoma (PEL) and multicentric Castleman’s disease (MCD) [1, 2, 3]. Whereas, EBV has been linked with multiple malignancies including Burkitt’s lymphoma (BL), Hodgkin’s lymphoma (HL), nasopharyngeal carcinoma (NPC) and gastric carcinoma (GC) [4, 5, 6].

Like all herpesviruses, KSHV and EBV have a biphasic life cycle comprising latent and lytic replication programmes. During latency, both viruses exist in a dormant state where only a subset of the viral genes are expressed facilitating the episomal persistence of the viral genome [7,8]. However, under certain physiological conditions, both viruses undergo lytic reactivation leading to expression of the full complement of lytic genes followed by the assembly and egress of infectious virions. Importantly however, both KSHV and EBV can also undergo abortive lytic reactivation, resulting in the expression of early lytic genes without subsequent virion assembly and cell lysis.

**Figure 1:**
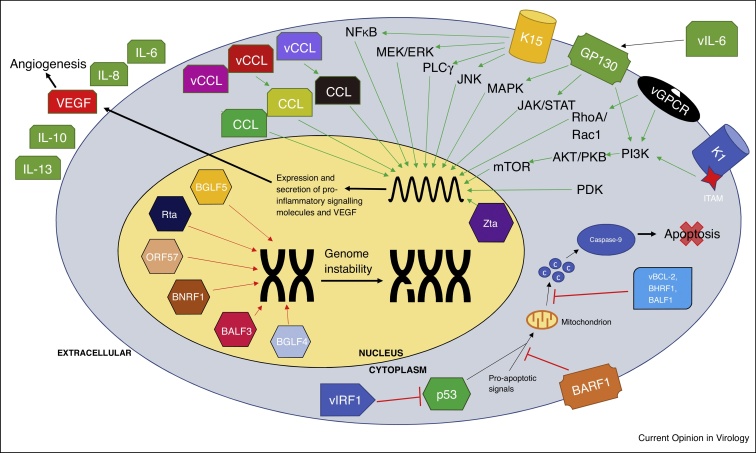
Schematic representation how KSHV and EBV lytically expressed proteins augment the pathogenesis of KSHV and EBV-associated malignancies.

Although much of the efforts to understand the molecular basis of these disorders has focused on viral latency, KSHV and EBV lytic cycles are now widely accepted as major contributors to oncogenesis which could be important targets in the development of anti-cancer therapeutics [9,10]. Thus, in this review, we discuss how lytic replication augments the pathogenesis of KSHV and EBV-associated malignancies (Figure 1) and the treatments available which may target the lytic replication cycle.

## KSHV lytic factors and tumourigenesis

Expression of the KSHV major lytic transactivator, RTA, is sufficient and necessary to activate the KSHV lytic cycle leading to a triphasic transcriptional cascade of immediate early, delayed early and late gene expression [11,12]. To initiate the transition from latency to lytic replication, a range of stimuli have been implicated including hypoxia, co-infection with HIV-1, oxidative stress and inflammatory cytokines; all of which promote the expression of RTA [13, 14, 15, 16]. Importantly, the treatment of KS patients with drugs that prevent lytic replication can, in certain cases, lead to regression of KS lesions; attesting to the importance of lytic gene expression in tumourigenesis [17].

Although *in vivo*, the spontaneous reactivation of KSHV takes place in only 1–3% of infected cells, the resultant assembly and egress of KSHV infectious particles sustains the population of latently infected cells that would otherwise be lost due to a combination of defects in episome synthesis during cell division and the death of infected cells [18]. Therefore, the balance between KSHV latent and lytic replication programmes is stringently controlled to ensure viral persistence and consequently tumour development. Nevertheless, in addition to the role of the lytic cycle in supporting a lifelong, persistent KSHV infection, a number of lytic factors themselves have oncogenic properties [19] (Table 1). Thus, the following section will outline these oncogenic lytic genes and discuss how they contribute to malignancy alongside their role in viral replication.

**Table 1:** KSHV lytic oncogenes and mechanisms of tumorigenesis

KSHV lytic gene	Cellular homologue	Lytic function	Mechanisms of oncogenesis	Oncogenic function	Reference
vGPRCR	IL-8 receptor	Activates cellular signalling pathways to maintain Rta expression	Secretes paracrine signalling molecules such as VEGF	Cell survival, angiogenesis	[22, 23, 24, 25,28]
			Activates Rho and Rac1 GTPases		
			Stimulates PI3K, PDK, AKT/PKB, p38 and MAPK signalling pathways		
K1	BCR	Activates cellular signalling pathways to maintain Rta expression	Contributes to VEGF autocrine and paracrine signalling	Cell survival, angiogenesis	[31, 32, 33, 34, 35,36^•^]
			Perturbs normal PI3K and MAPK signalling		
			Activates AKT and mTOR		
			Interacts with HIV-1 Tat to activate NF-κB		
K15		Activates cellular signalling pathways	Modulates PLCγ-Calcineurin-NFAT pathways	Angiogenesis	[37,38]
			Chemokine and cytokine production		
vIL-6	IL-6	Immune evasion and activation of cellular signalling pathways	Activates JAK/STAT, MAPK and PI3K/AKT pathways	Proliferation and angiogenesis	[40, 41, 42, 43]
			Contributes to VEGF autocrine and paracrine signalling		
vIRFs	IRFs	Immunoevasins	Inhibits Interferon α/β/γ responses and inflammatory signalling	Cell survival	[39,44, 45, 46, 47]
			Interaction with p53 prevents ATM/p53 DNA damage response pathway		
vBCL-2	BCL-2	Delays cell death	Inhibits apoptosis and autophagy	Cell survival	[48, 49, 50,51^•^,^52^]
vCCLs	CCLs	Immunoevasins	Modulation of CCL activity	Angiogenesis	[53, 54, 55]
			Chemoattractants for TH2 cells to reduce TH1 cell activity		
RTA		Lytic transactivator	Induction of DNA damage	Genome instability	[60]
ORF57		Processing, export and translation of viral RNAs	Sequesters the transcription and export complex (hTREX) to cause R-loop formation and DSBs	Genome instability	[57,58,59^•^]

## vGPCR

Viral G-protein-coupled-receptor (vGPCR) is an early lytic protein encoded by the viral gene ORF74. It shares limited homology to the human interleukin-8 receptors CXCR1 and CXCR2; however, ORF74 is constitutively active and minimally responsive to various ligands [20,21]. vGPCR activates members of the G protein family which stimulate several major cell signalling pathways such as phosphatidylinositol 3-kinase (PI3K), phosphoinoside-dependent kinase (PDK) and AKT/protein kinase B (AKT/PKB) along with several small GTPase proteins such as RhoA and Rac1 [22, 23, 24, 25]. The induction of a myriad of signalling pathways which activate Sp1/3 transcription factors is crucial to the maintenance of RTA expression and therefore commits the cell to productive lytic replication [26,27]. Previous studies have demonstrated that vGPCR is an oncogene capable of inducing angioproliferative lesions in transgenic mice that bear the pathological indicators of KS [28]. The angioproliferative nature of vGPCR appears to stem from its indirect production and secretion of a number of paracrine signalling molecules such as vascular endothelial growth factor (VEGF) [22, 23, 24, 25]. As a result, it has been suggested that vGPCR initiates the immortalisation of endothelial cells and KS tumourigenesis through the establishment of a VEGF paracrine loop [28]. In this proposed ‘hit and run’ model of immortalisation, populations of lytically replicating endothelial cells express vGPCR leading to VEGF expression and secretion. This acts in an autocrine mechanism on lytically replicating cells but also in a paracrine fashion on adjacent latently infected cells to promote their survival and immortalisation.

## K1 and K15

ORF-K1 encodes a type 1 transmembrane glycoprotein which shares structural similarities with the B cell receptor (BCR) [29]. The lytic protein appears to contribute to KSHV lytic replication in a similar manner to vGPCR through the maintenance of RTA expression; however, the specific mechanism behind this is not well understood [30]. Like vGPCR, K1 expression also contributes to the VEGF autocrine and paracrine signalling loop through its constitutively active ITAM domain, promoting the production and secretion of VEGF via matrix metalloprotease 9 (MMP-9) [31, 32, 33]. Increased VEGF signalling modulates downstream pathways such as PI3K and mitogen activated kinase which in turn activate Akt kinase and mammalian target of rapamycin (mTOR) to promote cell survival [32,34,35]. Furthermore, K1 has also been shown to interact with the HIV-1 protein Tat. Mouse studies have shown that coexpression of K1 and HIV-1 Tat leads to a synergistic increase in angiogenesis through the Tat-mediated upregulation of host microRNA miR-891a-5b which targets NF-κβ [36^•^].

Like ORF-K1, KSHV ORF-K15 encodes a transmembrane receptor which antagonises BCR signalling; however, K15 lack an ITAM domain and activates the production of cytokines and chemokines via cellular signalling pathways including NF-κB, MEK/ERK and Jun N-terminal protein kinase (JNK) pathways [37]. K15 bypasses the induction of VEGF signalling by activating the downstream PLCγ-Calcineurin-NFAT pathways to facilitate angiogenic tube formation in cell culture [38]. It is speculated that K15 is involved in the early development of KS tumour where limited lytic gene expression is detected [38].

## KSHV immunoevasins

Successful KSHV lytic replication is dependent on the expression of viral interferon regulatory factors (IRFs) and viral interleukins (IL) which prevent immune detection [39]. Most of these viral proteins are expressed during lytic replication to inhibit the interferon (IFN) antiviral response. KSHV vIL-6, like its human homologue can bind the gp130 receptor and activate the JAK/STAT, MAPK and PI3K/Akt cellular signalling pathways leading to expression of hIL-6 and VEGF [40, 41, 42]. Furthermore, the inoculation of NIH3T3 cells ectopically expressing vIL-6 into immunocompromised mice leads to tumour formation and vIL-6 expression in endothelial cells leads to angioproliferation and tubule formation [43].

The KSHV genome also possesses three lytically encoded vIRF ORFs: ORF-K9, ORF-K11/K11.1 and ORF-K10 encoding vIRF1, vIRF2 and vIRF4 respectively [39,44]. vIRFs act to disrupt the antiviral IFN response by inhibiting transcription of IFN-α/-β/-γ and inflammatory signals. The mechanism of vIRF action varies between isoforms; however, in many cases vIRF bind to cellular IRFs and inhibit their ability to activate transcription [39]. Through the dysregulation of the IFN antiviral response, apoptosis and cell cycle arrest are prevented increasing the oncogenic potential of KSHV-infected cells [44,45]. vIRF1 co-precipitates with p53, reducing p53 target gene expression [46]. Studies have demonstrated this vIRF1-p53 is crucial for the inhibition of the ATM/p53 DNA damage response pathway allowing viral DNA replication to proceed [39,46,47]. vIRF1 appears to block activation of ATM and decreases p53 stability through reduced phosphorylation of Ser15 of p53 [47].

## vBcl-2

vBcl-2, encoded by KSHV ORF16 shares sequence and functional homology to the Bcl-2 family of cellular proteins [48]. Normally, Bcl-2 proteins act as regulators of apoptosis and are characterised by four conserved stretches of amino acids called Bcl-2 homology (BH) domains [49]. Cellular Bcl-2 also negatively regulates autophagy by interacting with autophagy promoting factor Beclin-2 [50]. Studies in the murine counterpart of KSHV, murine herpesvirus 68 (MHV68), have shown that vBcl-2 is involved in both the inhibition of autophagy and apoptosis to promote B cell survival [51^•^]. The importance of autophagy in programmed cell death makes it an important target for KSHV during lytic infection to prevent induction of apoptosis and thus the impairment of viral replication. In KSHV the function of vBcl-2 has not yet been fully elucidated; however, lack of vBcl-2 impairs KSHV reactivation [48,52].

## vCCLs

KSHV also encodes three homologues of cellular chemokines known as viral CC-chemokine ligands (vCCLs) [53]. Previous studies have shown that vCCL1–vCCL3 are all able to bind to their cellular homologues both agonistically and antagonistically to stimulate angiogenesis [53,54]. Furthermore, all three viral proteins have been suggested to act as chemoattractants to modulate the levels of different T-cell subpopulations in KS lesions. The resultant TH2 cell-predominant tumour microenvironment in which TH1 cell responses are downregulated allowing immune evasion and thus tumour progression [55].

## KSHV and genome instability

KSHV lytic replication is associated with the formation of double strand-breaks (DSBs) and chromosomal aberrations, which are a common feature of KS lesions [56]. The KSHV early protein ORF57 controls the processing, export and translation of viral RNAs [57,58]. However, studies have shown that sequestration of the human transcription and export complex (hTREX) by ORF57 leads to DSBs as a result of R-loop formation [59^•^]. Furthermore, studies have suggested that Rta is also able to induce DNA damage [60]. Importantly, the activation of members of the DNA damage response machinery in this way has been suggested to facilitate viral DNA synthesis during productive lytic replication; as is the case with other Herpesviruses [60]. Thus, the role of KSHV lytic factors in genome instability likely contributes to oncogenesis.

## EBV lytic factors and tumourigenesis

EBV establishes a lifelong infection in B lymphocytes achieved through a highly regulated viral gene expression program. In latently infected B cells, the expression of either Zta or Rta is sufficient to reactivate the EBV lytic cycle [61, 62, 63, 64]. Lytic replication can be studied by treating latently infected B cells with inducers of the lytic cycle such as phorbal esters or by crosslinking B cell receptors with anti-immunoglobulins [65,66]. There have been several important insights regarding the contribution of the EBV lytic cycle for virus-induced tumorigenesis *in vivo*. Mouse models have demonstrated that lytic replication incompetent-EBV particles are impaired in their ability to cause lymphomagenesis compared to wild type virus, despite similar infection levels [67]. Furthermore, acyclovir treatment, which blocks lytic viral genome replication but not lytic gene expression, is unable to prevent EBV associated lymphomagenesis; reinforcing the role of lytic cycle-induced paracrine signals in disease progression [68]. Finally, *in vivo* studies have shown that in KSHV-infected PEL cells, tumour formation is enhanced upon coinfection with EBV [69^••^]. Together, these studies strongly support the view that lytic gene expression is important for tumour progression, and that paracrine signals play an essential role.

Similar to KSHV and the role of lytic reactivation in KS, reactivation of EBV may aid transmission of the virus within the tumor microenvironment to establish latency and drive cellular proliferation. However, the likely predominant role of the EBV lytic cycle is to provide the necessary paracrine, anti-apoptotic and immunomodulatory signals required for tumorigenesis (Table 2).

**Table 2:** EBV lytic oncogenes and mechanisms of tumorigenesis

EBV lytic gene	Cellular homologue	Lytic function	Mechanisms of oncogenesis	Oncogenic function	Reference
ZTA		Lytic transactivator	Promotes secretion of proangiogenic factors IL-6, IL-8, IL-10, IL-13 and VEGF	Angiogenesis	[67,68,70,71]
BHRF1 and BALF1	BCL-2	Delay cell death	Inhibition of apoptosis	Cell survival	[73,74]
BILF1		Immunoevasin	Unknown but detected in EBV tumours		[82,83]
BNFR1		Nucleocapsid trafficking	Interacts with centromeres to cause centrosome overduplication	Genome instability	[85^••^]
BALF3	Terminase	DNA synthesis and incorporation into virons	DNA damage	Genome instability	[88]
BGLF4		Viral DNA replication and nuclear import of viral proteins	DNA damage	Genome instability	[86]
BGLF5		Host cell shut-off	DNA damage	Genome instability	[79, 80, 81,87]
BARF1	C-fms receptor	Immunomodulator	Modulates expression genes involved in apoptotic signalling	Cell survival	[76,77]
BCRF1	IL-10	Immunoevasin	Downregulates interferon γ	Cell survival	[75]

## ZTA

Various studies have shown that the expression of some lytic antigens alone is sufficient to induce the expression of immunomodulatory and paracrine factors associated with oncogenesis. The lytic transactivator Zta facilitates the secretion of IL-6, IL-8, IL-10 and IL-13 in addition to proangiogenic proteins such as vascular endothelial growth factor (VEGF) [67,68,70]. Soluble Zta has also been detected in the sera of post-transplant lymphoproliferative disease (PTLD) patients providing further evidence of a transformative role for this lytic protein [70]. Finally, Zta alone can downregulate the expression of CIITA, an essential transcription factor important for HLA-II expression permitting immune evasion and tumour progression [71].

## EBV and anti-apoptotic signalling

In addition to the paracrine effect, some lytic proteins elicit strong anti-apoptotic signals [72]. Like KSHV vBcl-2, the EBV lytic cycle-associated proteins BHRF1 and BALF1 are viral homologs of cellular Bcl-2 which perform anti-apoptotic functions critical for cellular transformation *in vitro* [73,74]. Similarly, BCRF1, which is analogous to cellular IL-10, increases the viability and transformation of EBV-infected B cells through downregulation of interferon-γ [75]. Finally, *BARF1* is one of the most highly expressed genes in NPC cell lines and antibodies are frequently detected in NPC-patient sera [73]. The encoded protein, BARF1, a homolog of colony-stimulating factor 1 receptor, is a secreted anti-apoptotic factor which influences the survival of neighbouring cells [76,77]. Taken together, these studies implicate anti-apoptotic signalling by EBV lytic proteins in the oncogenesis of EBV-associated malignancies. However, although the expression of BHRF1, BALF1 and BARF1 is dramatically increased during lytic reactivation, and they have previously been designated as lytic genes, their expression has been detected in LCLs (lymphoblastoid cell lines) where cells are predominantly latently infected (>95%) [78]. Furthermore, BHRF1 is expressed from the latent promoter Wp in a subset of BL known as Wp-restricted BL [78]. Therefore, it is unclear whether these proteins contribute to tumorigenesis during the latent or lytic life cycles.

## BGLF5 and BILF1 immunoevasins

Several EBV lytic proteins, which primarily function as immunoevasins, also contribute to tumorigenesis. BGLF5, the EBV host shut-off protein which inhibits translation of host mRNAs, functions in the downregulation of toll-like receptor 9 (TLR9) and human leukocyte antigen class I (HLA-I) and –II leading to impaired T cell recognition [79,80]. Importantly however, BGLF5 expression has been detected in NPC biopsies and BGLF5 antibodies have been detected in NPC patient sera, suggesting the protein also undertakes a transformative role [81]. Similarly, the lytic EBV immunoevasin BILF1 enhances the internalisation of surface molecules of HLA-I leading to their rapid degradation by the lysosome; again impairing T cell recognition [82]. However, BILF1 expression has also been detected in NPC cells, once again suggesting an oncogenic function [83].

## EBV and genomic instability

Like KSHV, several EBV lytic proteins have also been implicated as contributors to genomic instability [84]. A recent study suggested that the EBV major tegument protein, BNFR1, involved in translocation of the viral nucleocapsid to the nucleus, induces centrosome amplification and thus contributes to the accumulation of chromosomal aberrations in infected cells [85^••^]. Furthermore, BNFR1 was able to induce genomic instability within latently infected LCLs without necessarily establishing infection, demonstrating that EBV lytic replication can promote transformation in adjacent cells [85^••^]. Three further EBV proteins, BALF3, BGLF4 and host cell-shutoff protein BGLF5 have all been suggested to further contribute to chromosomal instability and tumourigenesis through induction of DNA damage in NPC cells [86, 87, 88]. Thus, genomic instability appears a major mechanism through which EBV lytic proteins contribute to oncogenesis.

## Treatments for KSHV and EBV-associated cancers

Considerable advances in the understanding of the KSHV and EBV life cycle and related pathologies have been made since their discovery, however, presently there are still no vaccines or effective direct therapeutic options available for the prevention or treatment of their associated cancers. Almost all clinically available therapies that target the lytic life cycle of KSHV and EBV do not directly inhibit the virus and have shown varying results in the clinic (Table 3). Because of the critical role of lytic replication in disease progression and virus dissemination these highlight the need for potent and selective therapeutics against lytic viral targets to treat KSHV and EBV-associated cancers.

**Table 3:** Current treatments and inhibitors targeting the lytic life cycles of KSHV and EBV-associated cancers. Stage of development abbreviations: R = Randomised, C = Control and SP = Single Patient. Clinical disease abbreviations: HSV = Herpes simplex virus, VZV = Varicella zoster virus, CMV = Cytomegalovirus, HBV = Hepatitis B virus, ML = Myeloid leukaemia, ALL = Acute lymphoblastic leukaemia, LAM = Lymphangioleiomyomatosis, MDS = Myelodysplastic syndromes, CMML = Chronic myelomonocytic leukaemia and CTCL = Cutaneous T-cell lymphoma

Therpay target	Inhibitor class	Inhibitor subclass	Drug	Stage of development		Already clinically approved for:
				KSHV	EBV	
Viral DNA polymerase	Nucleoside analogues	Purine analogues	Acyclovir	Cohort study	*In vitro*	HSV and VZV
			Valganciclovir	R, C trial	SP study	CMV
		Pyrimidine analogs	Zidovudine	R trial	*In vivo*	HIV
		Methylenecyclopropane nucleosides	Cyclopropavir	*In vitro*	*In vitro*	None
	Acyclic nucleoside phosphonates	HPMP derivatives	Cidofovir	Pilot study	*In vivo*	CMV
		PME derivatives	Adefovir	*In vitro*	*In vitro*	HBV
	Pyrophosphate analog		Foscarnet sodium	Cohort study	*In vitro*	CMV
	Non-nucleoside inhibitor	Pyrimidoquinoline analog	NSC 373989	*In vitro*	None	None
Viral mRNAs	Peptide-conjugated phosphorodiamidate morpholin oligomers (PPMO)		PPMO against RTA	*In vivo*	n/a	None
			PPMO against vIRF-1	*In vitro*	n/a	None
Viral capsid protease	Small molecule helical mimetic	Dimerisation inhibitor	DD2	*In vitro*	*In vitro*	None
Lytic repression	Small-molecule inhibitors	Stilbenoid	Resveratrol	*In vitro*	*In vitro*	Dietary supplement
		Lipid	Delta-9 tetrahydrocannabinol	*In vitro*	*In vitro*	Multiple sclerosis
Ephrin receptor tyrosine kinase A2	Small-molecule inhibitors	ATP-competitive tyrosine kinase inhibitor	Dasatinib	*In vitro*	Phase I trial	Chronic ML and ALL
			4-(2,5-dimethyl-pyrrol-1-yl)-2-hydroxy-benzoic acid	*In vitro*	None	None
Dihydroorotate dehydrogenase/p53	Small-molecule inhibitor		Teriflunomide	None	*In vitro*	Multiple sclerosis
HSP70	Small-molecule inhibitor	ATP-derivative inhibitor	VER-155008	*In vitro*	None	None
hTREX	Small-molecule inhibitor	ATPase inhibitor	CCT018159	*In vitro*	None	None
mTOR	Polyketide		Rapamycin	Phase I trial	*In vivo*	Organ transplant rejection and LAM
Lytic induction	Cytidine analogue	DNA demethylation agent	5-azacytidine	*In vitro*	*In vitro*	MDS, Acute ML and CMML
	Small-molecule inhibitor	N-protected dipeptide	Bortezomib	*In vivo*	*In vitro*	Multiple myeloma
		Neddylation inhibitor	Pevonedistat	*In vitro*	*In vitro*	None
		Lipid	Valproic acid	*In vitro*	Pilot study	Anticonvulsant
		Histone deacetylase inhibitor	Suberoylanilide hydroxamic acid	*In vivo*	*In vivo*	CTCL
			C7	None	*In vitro*	None
Protective T-cell immunity	Vaccine	Capsid protein	gp350	n/a	Phase II trial	None
		Replication-competent viruses	Latency deficient replication-competent viruses	*In vivo*	None	None
	Cytokine therapy		GM-CSF and IL-2	None	*In vivo*	None

There is no standard of care for the treatment of KSHV-associated tumours and current options range from targeting cancers through surgical excision, chemotherapy and radiotherapy [89]. These treatment options are also recommended for EBV-associated cancers however, due to the array of cancers associated with EBV, treatment guidelines vary greatly for each different associated cancer [90].

To date, the most effective treatment of AIDS-related KS and AIDS-related EBV-associated cancers is highly active antiretroviral therapy (HAART), which works mostly through restoration of the patient’s immune system [91]. Likewise, iatrogenic KS, MCD, PEL and iatrogenic EBV-associated sarcomas and lymphomas are treated through the removal of immunosuppressants, to restore the patient’s immune system, limiting tumour progression, however this in turn can lead to graft rejection [92].

Immunotherapies have also been demonstrated to be effective at treating KSHV and EBV-associated cancers. Rituximab (anti-CD20) is clinically approved for the treatment of many EBV-associated lymphoproliferative diseases and also MCD [93^••^,^94^]. In addition, Tocilizumab (anti-human IL-6 receptor) and Siltuximab (anti-IL6 chimeric monoclonal antibody) are clinically available for the treatment of MCD [95,96].

## Novel therapies involving lytic KSHV and EBV

The majority of cells present in KSHV and EBV-associated cancers are latently infected therefore, there is substantial research exploring the potential of lytic induction therapy to treat these cancers. This treatment involves the efficient induction of all latently infected tumour cells into the lytic cycle while concomitantly exposing the cells to inhibitors of lytic replication and inducing apoptosis to clear all virally infected cells. In addition, lytic induction therapy can help induce a cytotoxic T-lymphocyte (CTL) response to lytic antigens to further clear cancerous virally infected cells. Lytic induction therapy poses a powerful mechanism to enhance the efficacy of EBV and KSHV lytic inhibitors, which are discussed below.

Because of the critical role of the KSHV and EBV lytic life cycle in tumorigenesis there is considerable interest in developing vaccines that target lytic antigens [97]. Various vaccines have been created targeting a range of lytic KSHV and EBV antigens. Some of these vaccines have proved successful *in vivo* and in clinical trials, such as the EBV envelope protein gp350, which reduced primary infection however, the vaccine failed to decrease the overall EBV infection rate.

Finally, cytokine therapy can induced protective T-cell immunity against viruses. Cytokine therapy in a humanised mouse model with EBV-associated lymphoproliferative disease induced a marked expansion of Zta-specific T-cells, which can prolong survival [98^•^,^99^]. In addition to supporting the notion that Zta plays a critical role in lymphoproliferative disease, it provides a strong rationale for the inclusion of Zta-specific antigens in vaccine development [97].

## KSHV and EBV lytic inhibitors

The lytic life cycles of KSHV and EBV pose numerous attractive and viable targets for the development of anti-viral drugs. However, the only KSHV and EBV inhibitors clinically available (and all anti-herpesvirus drugs in general) target the viral DNA-polymerase [100]. The most common of these drugs are nucleoside analogues, but acyclic nucleoside phosphonates (ANPs) and pyrophosphate analogues are also frequently used for herpesvirus therapy [89]. Clinically available nucleoside analogues are administered as pro-drugs and only activated by the viral thymidine kinase present during lytic replication [101]. Novel viral DNA-polymerase inhibitors have also been developed such as, second and third generation, nucleoside analogues and ANPs, and non-nucleoside inhibitors [102,103].

Many other inhibitors of herpesvirus replication have also been explored which target numerous aspects of the lytic life cycle, however none have made it as yet into the clinic. Targets with inhibitors demonstrated to have efficacy against EBV and KSHV include, the KSHV latent-lytic transactivator RTA, KSHV IRFs and the viral capsid protease [104,105].

Cellular targets required for lytic reactivation of EBV and KSHV and that contribute to lytic EBV and KSHV-associated tumorigenesis have also been approached as targets for inhibitors of their associated cancers [106]. Cellular inhibitors bear an increased risk of cytotoxic side effects, however, they possess great advantages due to fewer occurrences of drug-resistance and the potential for a broader activity against a range of viruses. Targets include, the KSHV/EBV cellular entry receptor (ephrin receptor tyrosine kinase A2), the proteasome, Hsp70, the human transcription/export complex (hTREX), the mammalian target of rapamycin (mTOR) and the dihydroorotate dehydrogenase enzyme [106,107^•^,^108^,109^•^,^110^,^111^]. While most inhibitors of cellular targets have only been demonstrated *in vitro* some have also shown efficacy in the clinic.

All clinically available inhibitors of EBV and KSHV have low efficacy and only target the viral DNA polymerase. Therefore, more efforts should be invested to examine the potential of drugs that target other viral proteins, since this proof-of-principle has been shown beneficial for other herpesviruses, such as HSV and HCMV.

## References and recommended reading

Papers of particular interest, published within the period of review, have been highlighted as

• of special interest
•• of outstanding interest
